# Optimizing the control group for evaluating ART outcomes: can outpatient claims data yield a better control group?

**DOI:** 10.1007/s10815-021-02111-6

**Published:** 2021-02-19

**Authors:** Judy E. Stern, Chia-Ling Liu, Xiaohui Cui, Daksha Gopal, Howard J. Cabral, Charles C. Coddington, Stacey A. Missmer, Sunah S. Hwang, Leslie V. Farland, Dmitry Dukhovny, Hafsatou Diop

**Affiliations:** 1 Department of Obstetrics and Gynecology and Pathology, Dartmouth-Hitchcock, Lebanon, NH 03756 USA; 2 Massachusetts Department of Public Health, Bureau of Family Health and Nutrition, Boston, MA USA; 3 Department of Biostatistics, Boston University School of Public Health, Boston, MA USA; 4 Department of Obstetrics and Gynecology, Carolinas Medical Center/Atrium Health, Charlotte, NC USA; 5 Department of Obstetrics, Gynecology, and Reproductive Biology, Michigan State University, East Lansing, MI USA; 6 Department of Pediatrics, University of Colorado School of Medicine, Aurora, CO USA; 7 Department of Epidemiology and Biostatistics, Mel and Enid Zuckerman College of Public Health, University of Arizona, Tucson, AZ USA; 8 Department of Pediatrics, Oregon Health & Science University, Portland, OR USA

**Keywords:** Assisted reproductive technology, APCD, ART outcomes, infertile, subfertile

## Abstract

**Purpose:**

We previously developed a subfertile comparison group with which to compare outcomes of assisted reproductive technology (ART) treatment. In this study, we evaluated whether insurance claims data in the Massachusetts All Payers Claims Database (APCD) defined a more appropriate comparison group.

**Methods:**

We used Massachusetts vital records of women who delivered between 2013 and 2017 on whom APCD data were available. ART deliveries were those linked to a national ART database. Deliveries were subfertile if fertility treatment was marked on the birth certificate, had prior hospitalization with ICD code for infertility, or prior fertility treatment. An infertile group included women with an APCD outpatient or inpatient ICD 9/10 infertility code prior to delivery. Fertile deliveries were none of the above. Demographics, health risks, and obstetric outcomes were compared among groups. Multivariable generalized estimating equations were used to calculate adjusted relative risk (aRR) and 95% confidence intervals (CI).

**Results:**

There were 70,726 fertile, 4,763 subfertile, 11,970 infertile, and 7,689 ART-treated deliveries. Only 3,297 deliveries were identified as both subfertile and infertile. Both subfertile and infertile were older, and had more education, chronic hypertension, and diabetes than the fertile group and less than the ART-treated group. Prematurity (aRR = 1.15–1.17) and birthweight (aRR = 1.10–1.21) were increased in all groups compared with the fertile group.

**Conclusion:**

Although the APCD allowed identification of more women than the previously defined subfertile categorization and allowed us to remove previously unidentified infertile women from the fertile group, it is not clear that it offered a clinically significantly improved comparison group.

**Supplementary Information:**

The online version contains supplementary material available at 10.1007/s10815-021-02111-6.

## Introduction

Assisted reproductive technology (ART) comprises the infertility treatment procedures in which a woman’s eggs are removed from her body and embryos are cultured in vitro [1]. Numerous studies over many years have shown that even singleton ART pregnancies result in an increase in adverse outcomes when compared with unassisted pregnancies to fertile women [2–4]. However, the question remains as to whether these adverse outcomes are a result of the ART procedures or underlying medical conditions associated with infertility [5]. To study this, a more appropriate comparison group, such as pregnancies conceived with other fertility treatments or those to women with infertility but no treatment, must be used. We have previously defined a comparison group that we called the “subfertile” group with which we compared outcomes following ART treatment [6]. We used the term subfertile for this group because it was a heterogenous group comprising pregnancies conceived with non-ART treatments identified from fertility treatments indicated on birth certificates, prior infertility diagnosis recorded on the hospital discharge records, and/or a history of prior ART or other fertility treatment but without necessarily having a defined diagnosis of infertility. Using this comparison group, we have found that adverse outcomes with ART were more similar to adverse outcomes in the subfertile group than the fertile group [7–9]. Although the subfertile group has been a useful comparison group, it is limited by the fact that we have no evidence that all of the women included have a defined diagnosis of infertility and from the likelihood that some women with infertility were missed and instead included in the fertile group.

Most infertility diagnosis and treatment is performed in an outpatient setting. Our prior studies of ART-treated and subfertile women used a linked database compiled from birth certificates, fetal death records, and hospital discharge records in the Pregnancy to Early Life Longitudinal (PELL) data system. PELL does not include outpatient data. Ascertaining women with infertility would be optimized if we could use outpatient records to identify women with an infertility diagnosis. One way to identify outpatient information is by using medical insurance claims data [10]. In Massachusetts, the Center for Health Information and Analysis (CHIA) has been provided with broad authority to collect healthcare claims data and to develop the All Payers Claims Database (APCD) under Massachusetts healthcare reform law. APCD collects claims data from insured patients in Massachusetts which are used by researchers to analyze population-level healthcare utilization and to determine quality outcomes for costs and pricing. The information in this system contains International Classification of Diseases (ICD) codes designated during outpatient as well as inpatient encounters to provide a supporting diagnosis for the visit.

In this study, we used the APCD to identify deliveries to women with a provider-defined insurance claim for the diagnosis of infertility but no ART treatment for that delivery, understanding that there would be some overlap between this group and the previously identified subfertile group (as defined above). We compared this group, which we defined as “infertile,” to our previously defined heterogeneous subfertile group, to a fertile group, and to an ART-treated group. Our goal was to evaluate whether this APCD-defined infertile group was a more complete, representative, and accurate comparison group than the subfertile group for outcomes to ART-treated women and to determine whether outcomes in this group were substantially different from those previously reported in the subfertile group.

## Materials and methods

### Data sources

We used data from (1) the Society for Assisted Reproductive Technology Clinic Outcome Reporting System (SART CORS), (2) the Massachusetts-based Pregnancy to Early Life Longitudinal (PELL) data system, and (3) the Massachusetts All Payers Claims Database (APCD). The study had approval from the Massachusetts Department of Public Health (MDPH) and the Dartmouth-Hitchcock Health Institutional Review Board. A Memorandum of Understanding was executed among SART, MDPH, and the project principal investigators.

The SART CORS database contains ART data entered by the clinics and reported to the Centers for Disease Control and Prevention in compliance with the Fertility Clinic Success Rate and Certification Act of 1992 (Public Law 102–493). The database contains ART cycle-specific demographics, infertility diagnoses, ART treatment, pregnancy, and outcome data, and is maintained by Redshift Technologies, Inc., under contract to SART. Data are obtained from approximately 90% of ART clinics in the USA and all Massachusetts clinics are included in the database. SART CORS data are validated annually with random clinics having on-site visits for chart review based on an algorithm for clinic selection. During these visits, data reported by the clinic are compared with information recorded in patients’ charts. In 2017, most data fields selected for validation were found to have discrepancy rates of ≤ 5% [11].

The PELL data system links Massachusetts birth certificates and fetal death records to corresponding hospital utilization data for the delivery event for the mother and infant, and to non-delivery hospital utilization (hospital admissions, observational stays, and emergency room visits) for the mother and child over time. The data have been linked for 98% of births and fetal deaths for individual women and their children since 1998. PELL data are linked through randomly generated unique IDs for mothers and infants. MDPH and CHIA are the custodians of the PELL data which are housed at MDPH.

The APCD is a comprehensive claims database that houses insurance claims from public and private insurance payers providing insurance to Massachusetts residents and employees. The database includes claims for medical, pharmacy, dental, vision, behavioral health, and specialty services. We obtained claims data for all available company and employer-sponsored insurance claims linked to women who delivered between January 1, 2013, and December 31, 2017. We are unaware of any validation studies on APCD data.

### Linkage of SART CORS to PELL

We developed the Massachusetts Outcome Study of Assisted Reproductive Technology (MOSART) database as previously defined [12] by linking the SART CORS and PELL data systems for all Massachusetts resident women delivering in Massachusetts hospitals for deliveries from 2004 to 2017. Linkage was performed using a deterministic five-phase linkage algorithm. Linkage was based on mother’s date of birth, her first name and last name, father/partner’s last name, baby’s date of birth, plurality, and infant sex. The linkage rate for 2004–2017 data was 91.5% overall and 94.9% for deliveries in which both mother’s zip code and clinic were located in Massachusetts.

### Linkage of MOSART to APCD

MOSART data from 2013 through 2017 were linked to the APCD under an MOU among CHIA, MDPH, and the project PIs. Information on PELL variables of the women and children from the MOSART database was submitted to CHIA for linkage using the member eligibility (ME) file. Variables included mother’s date of birth, last and first names, and zip codes for the women’s linkage, and infant’s date of birth, last and first names, sex, and zip codes for the child’s linkage. Upon obtaining the ME identifiers, CHIA matched and then extracted the APCD non-MassHealth medical claim (MC) records for the linked mothers and children and sent these data back to the PELL. Overall, 98.7% of the MOSART mothers and almost 100% of the MOSART children in 2013–2017 were linked to the APCD ME file of which 81% of the mothers and 54% of the children had at least one APCD entry. We did not have approval from MassHealth (the Massachusetts Medicaid provider) to obtain APCD insurance claims data, and thus although we linked all women in MOSART to APCD, the MassHealth claims were not included in these data. The 10.3% of companies that did not enter data into APCD could also not be included.

### Patients

The study sample included all deliveries for MOSART-APCD-linked women with no MassHealth records for October 1, 2013–December 31, 2017. Deliveries included those from October 1, 2013, rather than January of 2013 to allow us to have 9 months of APCD data in which to find an infertility code for that delivery in our dataset if one existed.

### Outcome measures

We obtained information on birthweight and gestational age from birth certificates. Clinically determined gestational age was modified, when needed, by reported dates of last menstrual period. Gestational ages outside of the range of 17–44 weeks were set as missing. Neonatal death was obtained from linked birth certificate and infant death data.

### Fertility groups

Deliveries were classified as ART-treated if the delivery was linked to an ART cycle in the SART CORS database. The subfertile group was defined as previously described [6] as having one or more of the following: (1) a marked checkbox for infertility treatment on the birth or death certificate, (2) an ICD9 or 10 code for infertility (ICD codes 628 and V230; ICD 10 O09.00-O09.03 and N97.0-N97.9) during a prior hospitalization, (3) prior delivery with either a checkbox for infertility treatment or linkage to SART CORS. A delivery was defined as infertile if the woman who delivered had an APCD outpatient or inpatient claim prior to that delivery with a provider-confirmed diagnosis of infertility (ICD codes as above). Women were classified as fertile if they did not fall into any of the other categories.

### Covariates

The following covariates were obtained from birth and death certificates: maternal and paternal age, race/ethnicity and education, maternal BMI, prior gravidity and parity, and infant sex. Information from birth, certificates, death certificates, and hospital discharge records was used to define: chronic hypertension and diabetes, gestational diabetes, pregnancy hypertension/preeclampsia/eclampsia, pregnancy-associated bleeding, and placental problems (abruptio placenta, placenta previa, vasa previa, and placenta accreta), other delivery complications including cephalopelvic disproportion, breech/malpresentation, prolonged labor, dysfunctional labor, febrile, fetal distress, cord prolapse, rupture membrane premature, rupture memberane prolonged, and caesarian hysterectomy, and method of delivery. APCD was used to define infertility diagnosis and treatment and SART CORS data for diagnosis was used for comparison to APCD in the ART group. We identified the following diagnoses related to infertility in the time period before the index delivery using ICD 9 and 10 codes for endometriosis, uterine, polycystic ovarian syndrome (PCOS), other ovulatory, diminished ovarian reserve (DOR), inflammatory conditions of the peritoneum and reproductive tract, and unexplained infertility (Supplemental Table 1). These diagnoses were also determined for the ART group in SART CORS using the reason for ART (rfa) fields. Treatment codes (Supplemental Table 1) were identified in the timeframe between LMP or presumptive LMP and delivery.

### Statistical analyses

Bivariate and multivariate generalizing estimating equations (GEE) with Poisson distribution and exchangeable correlation structure were used to account for multiple deliveries by the same women and to estimate relative risk ratios (RRs) and 95% confidence intervals (CIs). Models were adjusted for mother’s age (< 30, 31–34, 35–37, 38–40, > 40), race/ethnicity (Hispanic, NHW, NHB, NHA, NH-others, unknown), education (HS or < HS, some college, college, post college, unknown), chronic diabetes (yes, no), chronic hypertension (yes, no), parity (1, ≥ 2), gestational diabetes (yes, no), pregnancy hypertension including preeclampsia/eclampsia (yes, no), placental problems (yes, no), plurality (singleton, multiple), and infant gender (male, female). Analyses were performed in SAS software 14.3 (SAS Institute, Cary NC). In accordance with guidelines from MDPH, we suppressed any counts that were less than 11.

## Results

**Fig. 1:**
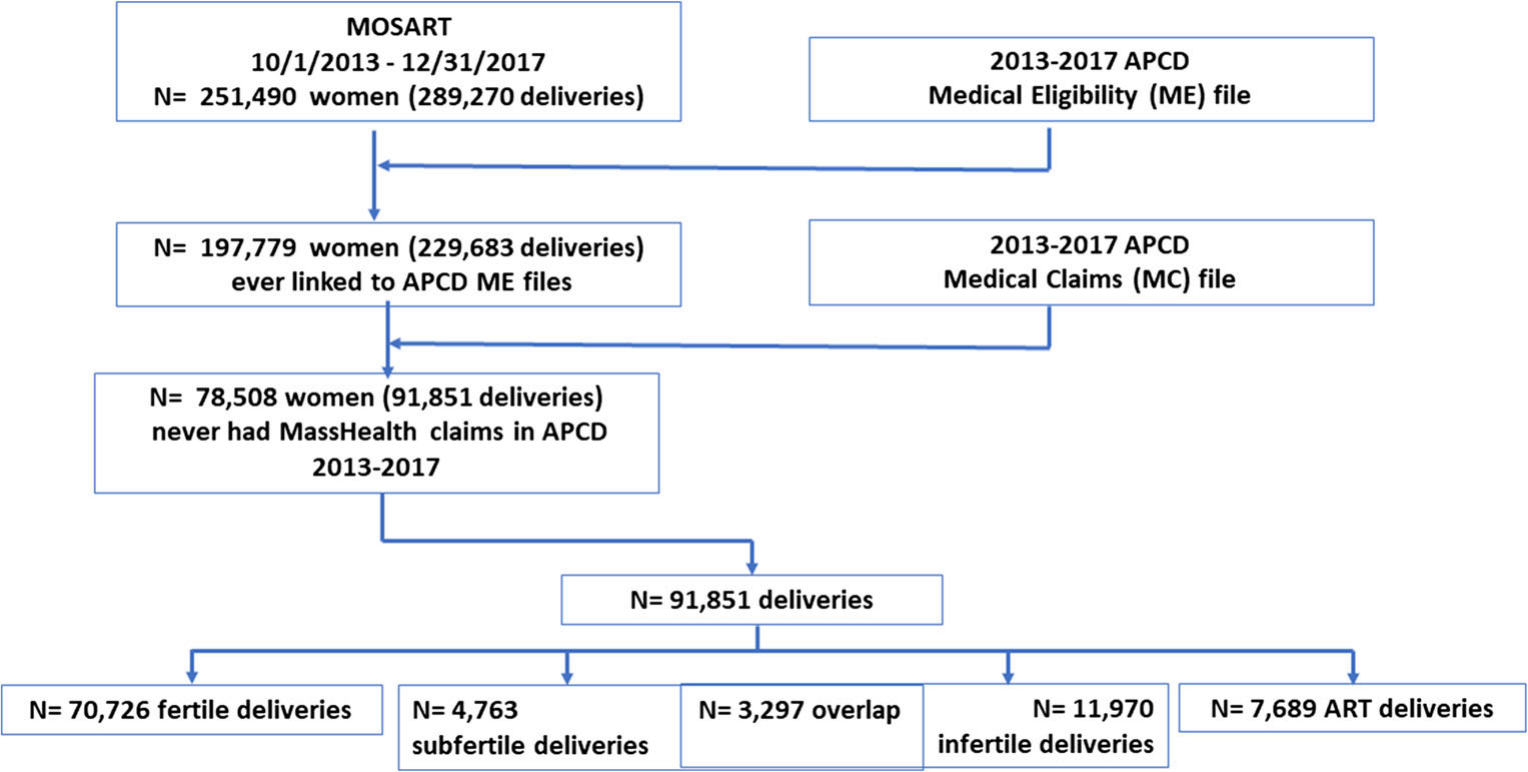
Study sample. Fertile deliveries are those not in any of the other groups; Subfertile deliveries are those to a woman who delivered had one or more of the following: a marked checkbox for infertility treatment on the birth or death certificate, an ICD9 or 10 code for infertility during a prior hospitalization, a prior delivery with either a checkbox for infertility treatment or linkage to SART CORS; infertile are deliveries to a woman with an APCD outpatient or inpatient claim prior to that delivery with a provider-confirmed diagnosis of infertility: ART deliveries were those linked to SART CORS

Our study sample included 91,851 deliveries to 78,508 women of which 70,726 were designated as fertile, 4,763 as subfertile, 11,970 as infertile, and 7,689 as ART-treated (Fig. 1). Only deliveries to women who did not have MassHealth at any time during the study period were included: this resulted in elimination of 60.0% of the deliveries (Fig. 1). More fertile (63.2%) than subfertile (39.2%), infertile (48.0%), or ART-treated (34.9%) deliveries were among those that were omitted due to those women having had any MassHealth during the study period.

The infertile cohort, defined as it was with the inclusion of outpatient data, contained close to 3 times as many deliveries as the previously defined subfertile group. Of those in the two groups, 2,406 (50.5%) in the subfertile group had a checkbox for fertility treatment marked on the birth certificate for the index delivery and 1,845 (15.4%) of the infertile group had this checkbox checked (Supplemental Table 2). Of the two cohorts, 1,466 were exclusively subfertile, 8,673 were exclusively infertile, and only 3,297 were identified as being in both groups. In addition, not all women identified in the subfertile group from the birth certificate (3,861 women) were identified through APCD (2,716, or 70.3%, of these women were identified in the infertile group). Of women who were ART-treated, 93.8% were identified as having an ICD 9 or 10 code for infertility in APCD prior to that delivery.

Table 1 compares the demographic characteristics of the 4 fertility groups. ART-treated, infertile, and subfertile women were older, more often white non-Hispanic, more highly educated, and more likely to be insured by private insurance at the time of delivery, than fertile women. Their partners were more likely to be older, white non-Hispanic, and highly educated. Women in these groups were also more likely to suffer from chronic hypertension and diabetes. With regard to the subfertile and infertile groups, both were younger, and both received more post-secondary education than the ART-treated group. Both the subfertile and infertile groups contained slightly more women who were white non-Hispanic than did the ART group. Overall, the infertile and subfertile groups were similar to each other. The use of ICD 9 and 10 codes to identify the infertile group resulted in more deliveries in that group than previously identified in the subfertile group. We therefore compared the demographics of the fertile group that we would have used as the control group if only the previously defined subfertile group was identified, to the fertile group containing only those women not in the three other groups. Results, shown in Supplemental Table 3, demonstrate that our previous inclusion of these extra deliveries (now known to include some infertile women) in the fertile group made very little difference in the characteristics of that group.

**Table 1:** Demographic characteristics in the four fertility groups

	Fertile^1^		Subfertile^2^		Infertile^3^		ART^4^	
	*n*	%	*n*	%	*n*	%	*n*	%
Total	70,726	100.00	4,763	100.00	11,970	100.00	7,689	100.00
Mother’s age								
Range	15–54		21–55		21–55		24–56	
Mean (SD)	32.51 (3.93)		34.84 (3.96)		34.47 (3.78)		36.24 (4.30)	
< 30	20,827	29.45	612	12.85	1,681	14.04	617	8.02
31–34	28,965	40.95	1,669	35.04	4,502	37.61	2,150	27.96
35–37	13,790	19.50	1,332	27.97	3,251	27.16	2,116	27.52
38–40	5,662	8.01	782	16.42	1,832	15.30	1,560	20.29
> 40	1,482	2.10	368	7.73	704	5.88	1,246	16.20
Mother’s race								
Hispanic	3,362	4.75	195	4.09	484	4.04	344	4.47
NHW	52,915	74.82	3,755	78.84	9,293	77.64	5,867	76.30
NHB	2,462	3.48	129	2.71	350	2.92	238	3.10
NHA	10,332	14.61	600	12.60	1,549	12.94	1,029	13.38
NH-others	312	0.44	15	0.31	50	0.42	31	0.40
Unknown	1,343	1.90	69	1.45	244	2.04	180	2.34
Mother’s education								
HS or < HS	2,493	3.52	106	2.23	294	2.46	154	2.00
Some college	9,033	12.77	478	10.04	1,211	10.12	749	9.74
College	26,501	37.47	1,632	34.26	4,175	34.88	2,615	34.01
Post college	31,011	43.85	2,463	51.71	5,982	49.97	3,949	51.36
Unknown	1,688	2.39	84	1.76	308	2.57	222	2.89
Father’s age								
Range	16-74		23-82		21-82		24-74	
Mean (SD)	34.52 (4.92)		36.68 (5.08)		36.31 (5.01)		38.18 (5.72)	
< 30	13,259	18.75	361	7.58	1,065	8.90	352	4.58
31–34	25,162	35.58	1,352	28.39	3,643	30.43	1,745	22.69
35–37	15,602	22.06	1,214	25.49	2,984	24.93	1,768	22.99
38–40	8,643	12.22	855	17.95	2,060	17.21	1,439	18.72
> 40	7,566	10.70	908	19.06	2,078	17.36	2,195	28.55
Unknown	494	0.70	73	1.53	140	1.17	190	2.47
Father’s race								
Hispanic	3,193	4.51	155	3.25	443	3.70	268	3.49
NHW	53,113	75.10	3,756	78.86	9,297	77.67	5,854	76.13
NHB	3,014	4.26	155	3.25	419	3.50	254	3.30
NHA	8,918	12.61	506	10.62	1,306	10.91	866	11.26
NH-others	455	0.64	29	0.61	80	0.67	41	0.53
Unknown	2,033	2.87	162	3.40	425	3.55	406	5.28
Father’s education								
HS or < HS	6,456	9.13	323	6.78	972	8.12	534	6.94
Some college	11,668	16.50	659	13.84	1,676	14.00	1,076	13.99
College	25,491	36.04	1,755	36.85	4,368	36.49	2,683	34.89
Post college	24,672	34.88	1,846	38.76	4,460	37.26	2,950	38.37
Unknown	2,439	3.45	180	3.78	494	4.13	446	5.80
Insurance at delivery								
Private	62,866	88.89	4,287	90.01	10,755	89.85	6,801	88.45
Free care	3,587	5.07	197	4.14	461	3.85	331	4.30
Self-pay	4,230	5.98	278	5.84	745	6.22	552	7.18
Unknown	43	0.06	< 11	--	< 11	--	< 11	--
Chronic conditions								
Hypertension	1,906	2.69	156	3.28	424	3.54	380	4.94
Diabetes	553	0.78	48	1.01	122	1.02	78	1.01
Mother’s BMI								
Underweight < 18.5	2,275	3.22	118	2.48	327	2.73	218	2.84
Normal weight 18.5–24.9	41,215	58.27	2,639	55.41	6,691	55.90	4,262	55.43
Overweight 25–29.9	15,649	22.13	1,102	23.14	2,715	22.68	1,725	22.43
Obese ≥ 30	9,434	13.34	772	16.21	1,861	15.55	1,197	15.57
Missing data	2,153	3.04	132	2.77	376	3.14	287	3.73
Gravidity								
1	28,027	39.63	1,321	27.73	4,215	35.21	3,100	40.32
2	23,771	33.61	1,612	33.84	3,954	33.03	2,355	30.63
> 2	18,652	26.37	1,814	38.09	3,742	31.26	2,203	28.65
Missing data	276	0.39	16	0.34	59	0.49	31	0.40
Parity								
1	33,862	47.88	1,792	37.62	5,939	49.62	4,624	60.14
2	26,260	37.13	2,017	42.35	4,621	38.60	2,465	32.06
> 2	10,523	14.88	950	19.95	1,395	11.65	594	7.73
Missing data	81	0.11	< 11	--	15	0.13	< 11	--
Plurality								
1	69,898	98.83	4,484	94.14	11,635	97.20	6,696	87.09
2	825	1.17	269	5.65	326	2.72	976	12.69
> 2	< 11	--	< 11	--	< 11	--	17	0.22

^1^Fertile: those deliveries not in any of the other groups

^2^Subertile: Deliveries to a woman with one or more of the following: a marked checkbox for infertility treatment on the birth or death certificate; an ICD9 or 10 code for infertility during a prior hospitalization; a prior delivery with either a checkbox for infertility treatment or linkage to SART CORS

^3^Infertile: Deliveries to a woman who had an APCD outpatient or inpatient claim prior to that delivery with a provider-confirmed diagnosis of infertility

^4^ART: The delivery was linked to SART CORS

We obtained information on infertility diagnoses from APCD by searching for any infertility code in the claims records in the time period prior to delivery. Table 2 shows the prevalence of these diagnoses in the various fertility groups. In the fertile group, only tubal disease (6.30%), PCOS (1.58%), and other ovulatory disorders (15.31%) were found at rates greater than 1% of the full sample. The percentage with no infertility diagnosis was approximately 73.4%. The proportion of women with all diagnoses was higher in the subfertile, infertile, and ART-treated groups than for the fertile group. Rates for the subfertile and infertile groups were similar to each other with some being slightly higher in the subfertile and some slightly higher in the infertile group, but rates for the ART-treated group were higher than either of the other groups for most diagnoses. Only the ovulatory diagnoses did not follow this pattern.

**Table 2:** Infertility diagnoses and treatment from APCD and SART CORS

Diagnoses and treatments determined from APCD claims (any visit before delivery)									Diagnoses determine from SART CORS	
	Fertile^1^		Subfertile^2^		Infertile^3^		ART^4^		ART^4^	
	*N*	%	*N*	%	*N*	%	*N*	%	*N*	%
Total	70,726	100.00	4,763	100.00	11,970	100.00	7,689	100.00	7,689	100.00
Endometriosis	556	0.79	143	3.00	455	3.80	537	6.98	413	5.37
Tubal	4,458	6.30	752	15.79	2,322	19.40	1,833	23.84	637	8.28
Uterine	58	0.08	92	1.93	290	2.42	423	5.50	188	2.45
PCOS	1,116	1.58	718	15.07	1,615	13.49	740	9.62	1,038	13.50
Other ovulatory	10,827	15.31	1,815	38.11	5,384	44.98	2,913	37.89	1,162	15.11
Diminished ovarian reserve	26	0.04	49	1.03	101	0.84	128	1.66	1,603	20.85
Inflammatory	201	0.28	91	1.91	262	2.19	413	5.37		
Male factor									2,467	32.08
Unexplained	0	0.00	2,920	61.31	10,411	86.98	6,516	84.74	2,028	26.38
None	51,902	73.38	1,205	25.30	558	4.66	755	9.82	538	7.00
Encounter for fertility treatment	63	0.09	420	8.82	842	7.03	4,788	62.27	7,689	100.00

^1^Fertile: those deliveries not in any of the other groups

^2^Subertile: Deliveries to a woman with one or more of the following: a marked checkbox for infertility treatment on the birth or death certificate; an ICD9 or 10 code for infertility during a prior hospitalization; a prior delivery with either a checkbox for infertility treatment or linkage to SART CORS

^3^Infertile: Deliveries to a woman who had an APCD outpatient or inpatient claim prior to that delivery with a provider-confirmed diagnosis of infertility

^4^ART: The delivery was linked to SART CORS

We further compared the diagnoses found in APCD to those reported by ART clinics to SART CORS. Here the percentages were very different with most being at lower rates in SART CORS than identified in APCD. By contrast, DOR was found at a much higher rate in SART CORS (20.85%) than in claims reported to APCD (1.66%). We were unable to identify male factor in APCD because our analysis was done on records for the females and that code would be found under the male partner’s records. Furthermore, 919 (12.74%) of the ART cohort with infertility in APCD had a diagnosis of Other in SART CORS (data not shown). Table 2 also presents information on claims data for fertility treatment codes. Of the ART patients who we know all had ART treatment as defined by linkage to SART CORS, a treatment code could only be found in 62.27% of cases in APCD. Codes for treatment could only be identified in 8.82% and 7.03% of the subfertile and infertile groups respectively.

Pregnancy and delivery characteristics for the four fertility groups are shown in Table 3. Women with ART-treated deliveries had consistently higher rates, and fertile deliveries had lower rates, of all adverse pregnancy and delivery complications including hypertension, diabetes, placental problems, dysfunctional labor, and post-delivery hysterectomy. As with other characteristics, the subfertile and infertile groups oscillated between which of them had the higher rate of various obstetric conditions, but both had lower rates than ART-treated and higher rates than the fertile cohort. The same pattern persisted for infant characteristics of low birthweight and prematurity (Table 4).

**Table 3:** Delivery characteristics in the four fertility groups

	Fertile^1^		Subfertile^2^		Infertile^3^		ART^4^	
	*n*	%	*n*	%	*n*	%	*n*	%
Total deliveries	70,726	100.00	4,763	100.00	11,970	100.00	7,689	100.00
Delivery method								
Vaginal	49,958	70.64	2,897	60.82	7,663	64.02	3970	51.63
Cesarean	20,759	29.35	1,865	39.16	4,307	35.98	3,717	48.34
Unknown	< 11	--	< 11	--	0	0.00	< 11	--
Pregnancy complications								
Pregnancy hypertension	4,974	7.03	341	7.16	933	7.79	776	10.09
Eclampsia/preeclampsia	3,020	4.27	286	6.00	659	5.51	718	9.34
Pregnancy diabetes	5,137	7.26	481	10.10	1,119	9.35	804	10.46
Delivery complications								
Abruptio placenta	898	1.27	89	1.87	205	1.71	176	2.29
Placenta previa	619	0.88	63	1.32	146	1.22	253	3.29
Vasa previa	32	0.05	< 11	--	17	0.14	35	0.46
Placenta accreta	244	0.34	25	0.52	56	0.47	74	0.96
Placental problems	1,712	2.42	165	3.46	385	3.22	487	6.33
Pregnancy/delivery bleeding problems	1,764	2.49	171	3.59	397	3.32	456	5.93
Cephalopelvic disproportion	1,131	1.60	55	1.15	190	1.59	128	1.66
Breech/malpresentation	7,156	10.12	605	12.70	1,469	12.27	1,429	18.58
Prolonged labor (> 20 h)	2,293	3.24	119	2.50	400	3.34	295	3.84
Dysfunctional labor	8,245	11.66	455	9.55	1,422	11.88	1,113	14.48
Febrile (GT 100f or 38c)	2,715	3.84	146	3.07	471	3.93	340	4.42
Fetal distress	6,651	9.40	387	8.13	1,082	9.04	691	8.99
Cord prolapse	236	0.33	14	0.29	45	0.38	33	0.43
PROM	4,530	6.40	295	6.19	782	6.53	600	7.80
Hysterectomy	57	0.08	11	0.23	27	0.23	34	0.44

^1^Fertile: those deliveries not in any of the other groups

^2^Subertile: Deliveries to a woman with one or more of the following: a marked checkbox for infertility treatment on the birth or death certificate; an ICD9 or 10 code for infertility during a prior hospitalization; a prior delivery with either a checkbox for infertility treatment or linkage to SART CORS

^3^Infertile: Deliveries to a woman who had an APCD outpatient or inpatient claim prior to that delivery with a provider-confirmed diagnosis of infertility

^4^ART: The delivery was linked to SART CORS

**Table 4:** Infant characteristics in the four fertility groups

	Fertile^1^		Subfertile^2^		Infertile^3^		ART^4^	
	*n*	%	*n*	%	*n*	%	*n*	%
Infant characteristics								
Total infants	71,556	100.00	5,052	100.00	12,314	100.00	8,699	100.00
Total live births	71,377	99.75	5,039	99.74	12,267	99.62	8,669	99.66
Gender								
Male	36,622	51.18	2,601	51.48	6,365	51.69	4,527	52.04
Female	34,931	48.82	2,451	48.52	5,949	48.31	4,171	47.95
Unknown	< 11	--	0	0.00	0	0.00	< 11	--
Birthweight								
Range	96–6575		113–5263		113–5470		155–5420	
Mean (SD)	3382.88 (546.40)		3268.87 (656.17)		3315.49 (612.67)		3124.99 (714.39)	
< 1,500gms	528	0.74	98	1.94	181	1.47	224	2.58
1,500–2,499gms	3,034	4.24	450	8.91	807	6.55	1,268	14.58
≥ 2,500gms	67,912	94.91	4,490	88.88	11,305	91.81	7,190	82.65
Unknown	82	0.11	14	0.28	21	0.17	17	0.20
Gestational age								
Range	17-44		19-44		19-44		18-43	
Mean (SD)	38.96 (1.73)		38.41 (2.26)		38.65 (2.07)		37.94 (2.61)	
< 32 weeks	469	0.66	94	1.86	165	1.34	242	2.78
32–36 weeks	3,794	5.30	532	10.53	986	8.01	1,492	17.15
≥37 weeks	66,891	93.48	4,382	86.74	11,055	89.78	6,857	78.83
Unknown	402	0.56	44	0.87	108	0.88	108	1.24
Neonatal deaths (live births only)								
0–7 days	92	0.13	15	0.30	26	0.21	37	0.43
7–28 days	19	0.03	< 11	--	< 11	--	< 11	--
29 days+	44	0.06	< 11	--	< 11	--	< 11	--
No death reported	71,222	99.53	5,018	99.33	12,224	99.27	8,622	99.11

^1^Fertile: those deliveries not in any of the other groups

^2^Subertile: Deliveries to a woman with one or more of the following: a marked checkbox for infertility treatment on the birth or death certificate; an ICD9 or 10 code for infertility during a prior hospitalization; a prior delivery with either a checkbox for infertility treatment or linkage to SART CORS

^3^Infertile: Deliveries to a woman who had an APCD outpatient or inpatient claim prior to that delivery with a provider-confirmed diagnosis of infertility

^4^ART: The delivery was linked to SART CORS

Table 5 presents risk ratios for low birthweight and preterm delivery for infants in the four fertility groups. The ART-treated as well as the subfertile and infertile groups had higher rates of prematurity (range of aRR was 1.15–1.17) and low birthweight (range of aRR 1.10–1.21) than the fertile group. When compared with the infertile group, the ART-treated group had a higher rate of preterm delivery (aRR 1.10) while the subfertile group did not differ. The ART-treated group did not differ from the subfertile group with regard to these parameters.

**Table 5:** Relative risk ratios for comparisons of four fertility groups

	Reference		Subfertile		Infertile		ART	
	*n*	%	RR (95% CI)^3^		RR (95% CI)^3^		RR (95% CI)^3^	
			Crude	Adjusted^4^	Crude	Adjusted^4^	Crude	Adjusted^4^
Reference fertile	Fertile							
Birthweight^1^								
LBW (< 2,500gms)	3,555	4.98	1.90 (1.71–2.10)	1.21 (1.07–1.34)	1.48 (1.37–1.61)	1.19 (1.10–1.28)	2.97 (2.78–3.17)	1.10 (1.01–1.20)
Gestational age^2^								
Preterm (< 37 weeks)	4,261	5.99	1.86 (1.68–2.06)	1.17 (1.06–1.29)	1.39 (1.28–1.50)	1.15 (1.07–1.24)	2.90 (2.72–3.10)	1.17 (1.09–1.27)
Reference infertile	Infertile							
Birthweight^1^								
LBW (< 2,500gms)	986	8.03	1.26 (1.01–1.58)	1.08 (0.95–1.22)	Reference	Reference	1.75 (1.48–2.08)	1.01 (0.93–1.10)
Gestational age^2^								
Preterm (< 37 weeks)	1,151	9.44	0.80 (0.24–2.69)	1.04 (0.93–1.17)	Reference	Reference	1.65 (1.23–2.22)	1.10 (1.01–1.19)
Reference subfertile	Subfertile							
Birthweight^1^								
LBW (< 2,500gms)	547	10.87	Reference	Reference			1.77 (1.27–2.47)	0.97 (0.88–1.07)
Gestational age^2^								
Preterm (< 37 weeks)	626	12.50	Reference	Reference			1.74 (1.30–2.34)	1.05 (0.96–1.15)

^1^Analysis excluded missing data on birthweight and parity

^2^Analysis excluded missing data on gestational age and parity

^3^Modified Poisson regression (GEE with Poisson distribution, exchangeable correlation structure) was used to account for multiple deliveries by the same women. Reference = Fertile group. Binomial-logarithm models would not converge/out of boundary limits

^4^RRs adjusted for mother’s age, race/ethnicity, education, chronic diabetes, chronic hypertension, parity, gestational diabetes, pregnancy hypertension including preeclampsia/eclampsia, placental problems, plurality, infant gender

## Discussion

This study identified a new cohort of deliveries to infertile women through analysis of claims data from the APCD. We compared this group to our previously identified heterogeneous subfertile group and to ART-treated and fertile groups. We found the infertile group defined by APCD to be considerably larger than the subfertile group but to have very similar characteristics. Some deliveries identified through one method were not identified by the other and vice versa.

It is tempting to use medical claims data for research. These data are extensive and have the potential to be tapped for a variety of research questions. Nevertheless, as previously reported [6, 13–17], the results in this paper suggest that caution is required when using these data. Claims data are only as good as the information entered by providers and the completeness of the insurance claims data file. In the case of the Massachusetts APCD, there were data missing from insurance providers who did not participate in the claims data upload. In MA, this was made more complicated by a 2016 lawsuit (https://www.chiamass.gov/assets/docs/p/apcd/regulatory-questions-for-apcds-related-to-scotus.pdf) that resulted in 10.3% of insurance providers opting to forego submitted data to the system from that point onwards (personal communication from CHIA). Although APCD uses a patient identifier to enable following each patient as she changes health insurance companies over time, patients could change in and out of those companies that do not enter data into the system. Thus, while we were able to obtain information on prior infertility for many patients, our numbers may still not be complete given that a small percentage of women may have had insurance with companies that did not enter data. Furthermore, the fact that 60% of women with deliveries had MassHealth, a Medicaid-based insurance option, during some point in the study period, resulted in our having no information on this group and thus our decision to remove these women from our study sample. This group with some MassHealth accounted for more fertile than subfertile, infertile, or ART-treated women likely because MassHealth does not cover infertility treatment and because of the demographics of women who seek fertility care, but this is still an omission. Regardless of these omissions, our study demonstrates that the infertile and subfertile groups both showed similar profiles for demographics and adverse pregnancy outcomes. Specifically, we observed that women in the infertile and subfertile groups had similar or lower risk of adverse pregnancy outcomes than those of ART-treated women but greater risk than the fertile group. This suggests that infertile and subfertile groups may be similar as far as a comparison group for studies of ART. As previously argued by us and others [5], this also suggests that underlying infertility is a factor contributing to the adverse outcomes seen following ART treatment.

The larger size of the infertile group suggests that many infertile deliveries were missed using the definitions by which we previously identified the subfertile group. Nevertheless, the similarities between the subfertile and infertile groups included demographic parameters, underlying health conditions, as well as pregnancy and delivery outcomes. In all cases, both the subfertile and the infertile groups had characteristics that were intermediate between those of the ART-treated and the fertile groups. We have acknowledged in prior publications that the subfertile comparison group likely did not contain all cases of infertility [7, 18, 19], which may lead to misclassification, and most likely previous results were attenuated due to this. We have also previously suggested that though the fertile group contained some deliveries to infertile women, those were likely subsumed within the much larger fertile cohort. The data presented in Supplemental Table 3 support these prior claims.

Only 3,297 deliveries overlapped and were contained in both the subfertile and infertile groups. The fact that these groups did not overlap more completely means that the subfertile group missed identifying a substantial proportion of deliveries for which a diagnosis of infertility had indeed been made. However, it also could mean that the subfertile group, being defined as it was in large part through the checkbox for fertility treatment on the birth certificate, included some individuals who had fertility treatment for reasons other than infertility such as being a single woman, a same-sex couple, or a couple undergoing fertility treatment for genetic conditions. This distinction cannot be determined from the birth certificates. The extent to which this is the case cannot be fully appreciated at this time; however, the fact that just under 13% of the ART cohort with infertility in APCD had an infertility diagnosis of “Other” in SART CORS suggests that we should not expect an infertility diagnosis in all these women. The designation of infertile also missed some deliveries found through the parameters of the subfertility definition. One reason for this is timeframe. Our APCD data only went back to January of 2013. By contrast, MOSART data used to identify subfertility included hospitalizations extending back to 1998. Cases of infertility identified in the subfertile group through hospital discharges before 2013 could thus have been missed when only APCD data were used. Furthermore, the APCD is based on insurance claims and is not a clinical database. The data in this system are dependent on how claims are processed and whether or not the woman had insurance provided by a participating medical insurance carrier. Although these data are valuable, they are not infallible or comprehensive, and as with other vital records and other non-medical databases, they are subject to limitations.

We have not previously been able to identify specific infertility-related diagnoses among the subfertile group and this is something that could be an advantage of using APCD. Analysis of infertility diagnosis and treatment from APCD yielded information on the reasons for infertility in all groups. Again, it was clear that the subfertile and infertile groups were similar and that the fertile group was less likely to have any infertility-related diagnosis. The ART-treated group had more women with various diagnoses than either the subfertile or infertile groups with a few notable exceptions on ovulation disorders potentially indicating that treatment was possible for these conditions without ART. When comparing to prior studies, the percentage of women identified as having endometriosis (< 4% in all but the ART group) was lower than prior 6–15% overall population estimates [20, 21], and the percentage of those with PCOS (< 5% of the population overall) also lower than the expected 6–10% in the general population [22]. Our estimates from APCD thus appear to have missed a proportion of the diagnosed cases. This will require further review and validation. The differences between the infertility diagnosis information obtained from APCD and that obtained from SART CORS is also of concern. In particular, the much higher rate of DOR identified in the SART CORS suggests that even when that diagnosis is given for an ART cycle, claims data do not necessarily include it. One reason for the differences may be that many clinics may enter only the primary diagnosis into SART CORS while review of ACPD would gather all the diagnoses from each clinic visit over time. Other reasons for this are at present unclear.

We also evaluated treatment codes in APCD to see the number of women on whom we could identify that fertility treatment was used for the index delivery. We found that these treatment codes, when found at all, were often identified outside of the timeframe that we anticipated. We had anticipated that most codes would be found within the timeframe in the month around oocyte retrieval or conception date. Instead, many were found closer to delivery date. Reasons for this are unknown. The fact that fewer women in the subfertile and infertile groups had claims for treatment than had an indication of fertility treatment on the birth certificate also suggests that either only a small proportion of them had intrauterine insemination or that many were treated with fertility drugs which were not part of our claims search. Although we have information on fertility medications in our APCD data, the claims information for this could not be temporally connected with a particular delivery since medication can be dispensed outside of a defined treatment window. This is another factor which makes insurance claims data particularly risky for use in studies of medical history and outcomes.

Risks of preterm delivery and low birthweight were elevated in our ART-treated, subfertile, and infertile groups when compared with the fertile group. This is consistent with prior results [3, 7]. The ART-treated group also had a higher adjusted rate of prematurity when compared with the infertile group but not the subfertile group (Table 5) perhaps reflecting the larger size and greater diversity of the infertile group compared with the subfertile. Importantly, the subfertile and infertile groups did not differ from each other in either prematurity or birthweight. The APCD-defined infertile group, therefore, was more similar to the subfertile group than to either the ART-treated or the fertile groups.

Our study has strengths and limitations. Among the strengths are the robust sample size in all groups and the availability of birth certificate and hospital data on underlying health and pregnancy complication information. In addition, we have the advantage of being able to have used two definitions of compromised fertility to approach the same questions on outcome. Limitations include reservations about the accuracy of APCD data and the fact that women with any MassHealth data had to be excluded. We also uncovered that the timing of claims in APCD in relationship to actual treatment dates was inconsistent with expectation and that this may be a limitation to future use of APCD claims data for research. A final limitation was that we used data from one state with a particularly comprehensive insurance claims file and thus our methodology and findings may not be generalizable or useful to investigators using databases from other states.

In summary, we defined a newly constituted infertile group based on information on infertility diagnosis in the APCD. This group was considerably larger than our previously defined subfertile group which was based on birth certificate data, on fertility treatment, and several other indicators of infertility. The advantage of this new infertile group is that all members of this group had a defined diagnosis of infertility. The disadvantage is that its use relies on claims data which must be considered to have a number of limitations as to accuracy and completeness. Both comparison groups (subfertile and infertile) had similar characteristics resulting in these groups being part way between those of the fertile and the ART-treated groups and both yielded similar, though not precisely the same, data on infant outcomes. In conclusion, we propose that each of these comparison groups, either separately or in combination, might have specific advantages for different study questions and that although both are better comparators than the fertile group, one is not considerably better or more unique than the other.

## Supplementary Information

ESM 1 (DOCX 25 kb)

## Data Availability

Access to data is limited due to restrictions placed by the Massachusetts Department of Public Health.

## Code availability

Access to software application or custom code is limited due to restrictions placed by the Massachusetts Department of Public Health.
