# Supplementary material for: Optimizing the control group for evaluating ART outcomes: can outpatient claims data yield a better control group?
Source: J Assist Reprod Genet. 2021 Feb 19;38(5):1089–100. doi: 10.1007/s10815-021-02111-6 (PMC8190220; doi:10.1007/s10815-021-02111-6)
Supplement: Supplementary file 1 — (DOCX 25 kb) [file 10815_2021_2111_MOESM1_ESM.docx]

Supplemental Table 1: ICD 9 and 10 codes for infertility diagnosis and treatment

| Diagnosis | ICD-9 codes | ICD-10 codes |
| --- | --- | --- |
| Endometriosis | 617.1, 617.2, 617.3, 617.8, 617.9 | N80.1 to N80.9 |
| tubal | 628.2, 620.0 | N97.1, N97.2 |
| uterine | 628.3, 628.4 | N84.0, N97.2 |
| PCOS | 256.4 | E28.2 |
| other ovulatory | 256.1, 256.2, 256.8, 256.9, 626.4, 626.8, 628.0 | E28.9 E28.8, E28.9, N97.0, N92.6 |
| Diminished ovarian reserve | 256.39 | E28.39 |
| Inflammatory | 614.0, 614.1, 614.2, 614.3, 614.4, 614.5, 614.8, 614.9, 628.3, 016.7 | N71.1-N71.9, N70.11, N70.01, N70.93, N73.9 |
| Male factor |  | Z31.81 |
| Unexplained | 628.9 | N97.9 |
| None (has none of the other conditions) |  |  |
| Encounter for fertility treatment (between presumed LMP and delivery) | V26.81, V26.1 | Z31.83, N98.0, 8E0ZXY1 HCPCS Code: S4020, S4021, S4011, S4014, S4015, S4017, S4022, S4023, S4037, S4035 |

Supplemental Table 2: How the infertile population compares with the population defined under the subfertile definition

| Subfertile defined | Subfertile | | Infertile that are also Subfertile | | Infertile All | |
| --- | --- | --- | --- | --- | --- | --- |
|  | N | % | N | % | N | % |
| Total | 4,763 | 100.0 | 3,297 | 100.0 | 11,970 | 100% |
| BC checkbox on the index delivery | 2,406 | 50.5% | 1,846 | 56.0% | 1,846 | 15.4% |
| BC checkbox on any delivery prior to or including the index delivery | 3,861 | 81.1% | 2,716 | 82.4% | 2,716 | 22.7% |
| By BC checkbox only | 2,922 | 61.3% | 2,059 | 62.5% | 2,059 | 17.2% |
| By prior ICD code only | 236 | 5.0% | 166 | 5.0% | 166 | 1.4% |
| By prior ART or BC only | 4,228 | 88.8% | 2,904 | 88.1% | 2,904 | 24.3% |
| BC and ICD code | 241 | 5.1% | 187 | 5.7% | 187 | 1.6% |
| BC and prior ART or BC | 3,861 | 81.1% | 2,716 | 82.4% | 2,716 | 22.7% |
| ICD and prior ART or BC | 3,919 | 82.3% | 2,756 | 83.6% | 2,756 | 23.0% |

BC= birth certificate

Supplemental Table 3: Comparison of the fertile population in this paper with that from prior papers

|  | Fertile without Subfertile, Infertile or ART | | Fertile without Subfertile or ART | |
| --- | --- | --- | --- | --- |
|  | n | % | n | % |
| Total | 70,726 | 100.00 | 79,399 | 100.00 |
| Mother's Age |  |  |  |  |
| Range | 15-54 | | 15-54 | |
| Mean (SD) | 32.51 (3.93) | | 32.71 (3.95) | |
| < 30 | 20,827 | 29.45 | 22,096 | 27.83 |
| 31-34 | 28,965 | 40.95 | 32,303 | 40.68 |
| 35-37 | 13,790 | 19.50 | 16,100 | 20.28 |
| 38-40 | 5,662 | 8.01 | 6,955 | 8.76 |
| > 40 | 1,482 | 2.10 | 1,945 | 2.45 |
| Mother's race |  |  |  |  |
| Hispanic | 3,362 | 4.75 | 3,722 | 4.69 |
| NHW | 52,915 | 74.82 | 59,609 | 75.08 |
| NHB | 2,462 | 3.48 | 2,721 | 3.43 |
| NHA | 10,332 | 14.61 | 11,459 | 14.43 |
| NH -Others | 312 | 0.44 | 350 | 0.44 |
| Unknown | 1,343 | 1.90 | 1,538 | 1.94 |
| Mother's Education |  |  |  |  |
| HS or < HS | 2,493 | 3.52 | 2,728 | 3.44 |
| Some College | 9,033 | 12.77 | 9,953 | 12.54 |
| College | 26,501 | 37.47 | 29,550 | 37.22 |
| Post college | 31,011 | 43.85 | 35,234 | 44.38 |
| Unknown | 1,688 | 2.39 | 1,934 | 2.44 |
| Father's Age |  |  |  |  |
| Range | 16-74 | | 16-74 | |
| Mean (SD) | 34.52 (4.92) | | 34.70 (4.96) | |
| <30 | 13,259 | 18.75 | 14,082 | 17.74 |
| 31-34 | 25,162 | 35.58 | 27,853 | 35.08 |
| 35-37 | 15,602 | 22.06 | 17,743 | 22.35 |
| 38-40 | 8,643 | 12.22 | 10,118 | 12.74 |
| >40 | 7,566 | 10.70 | 9,030 | 11.37 |
| Unknown | 494 | 0.70 | 573 | 0.72 |
| Father's race |  |  |  |  |
| Hispanic | 3,193 | 4.51 | 3,531 | 4.45 |
| NHW | 53,113 | 75.10 | 59,798 | 75.31 |
| NHB | 3,014 | 4.26 | 3,341 | 4.21 |
| NHA | 8,918 | 12.61 | 9,885 | 12.45 |
| NH -Others | 455 | 0.64 | 511 | 0.64 |
| Unknown | 2,033 | 2.87 | 2,333 | 2.94 |
| Father's Education |  |  |  |  |
| HS or < HS | 6,456 | 9.13 | 7,201 | 9.07 |
| Some College | 11,668 | 16.50 | 12,898 | 16.24 |
| College | 25,491 | 36.04 | 28,646 | 36.08 |
| Post college | 24,672 | 34.88 | 27,860 | 35.09 |
| Unknown | 2,439 | 3.45 | 2,794 | 3.52 |
| Insurance at Delivery |  |  |  |  |
| Private | 62,866 | 88.89 | 70,633 | 88.96 |
| Public/Free Care | 3,587 | 5.07 | 3,932 | 4.95 |
| Self Pay | 4,230 | 5.98 | 4,783 | 6.02 |
| Unknown | 43 | 0.06 | 51 | 0.06 |
| Chronic conditions |  |  |  |  |
| Hypertension | 1,906 | 2.69 | 2,206 | 2.78 |
| Diabetes | 553 | 0.78 | 641 | 0.81 |
| Mother's BMI |  |  |  |  |
| Underweight <18.5 | 2,275 | 3.22 | 2,526 | 3.18 |
| Normal weight 18.5–24.9 | 41,215 | 58.27 | 46,079 | 58.03 |
| Overweight 25–29.9 | 15,649 | 22.13 | 17,591 | 22.16 |
| Obesity ≥ 30 | 9,434 | 13.34 | 10,758 | 13.55 |
| Missing data | 2,153 | 3.04 | 2,445 | 3.08 |
| Gravidity |  |  |  |  |
| 1 | 28,027 | 39.63 | 31,240 | 39.35 |
| 2 | 23,771 | 33.61 | 26,583 | 33.48 |
| >2 | 18,652 | 26.37 | 21,253 | 26.77 |
| Missing data | 276 | 0.39 | 323 | 0.41 |
| Parity |  |  |  |  |
| 1 | 33,862 | 47.88 | 38,415 | 48.38 |
| 2 | 26,260 | 37.13 | 29,473 | 37.12 |
| >2 | 10,523 | 14.88 | 11,418 | 14.38 |
| Missing data | 81 | 0.11 | 93 | 0.12 |
| Plurality |  |  |  |  |
| 1 | 69,898 | 98.83 | 78,449 | 98.80 |
| 2 | 825 | 1.17 | 946 | 1.19 |
| >2 | <11 | -- | <11 | -- |
